# Effect of Glycine, Pyruvate, and Resveratrol on the Regeneration Process of Postischemic Intestinal Mucosa

**DOI:** 10.1155/2017/1072969

**Published:** 2017-10-19

**Authors:** Lisa Brencher, Frank Petrat, Katrin Stych, Tim Hamburger, Michael Kirsch

**Affiliations:** Institute of Physiological Chemistry, University Hospital Essen, University Duisburg-Essen, Essen, Germany

## Abstract

**Background:**

Intestinal ischemia is often caused by a malperfusion of the upper mesenteric artery. Since the intestinal mucosa is one of the most rapidly proliferating organs in human body, this tissue can partly regenerate itself after the onset of ischemia and reperfusion (I/R). Therefore, we investigated whether glycine, sodium pyruvate, and resveratrol can either support or potentially harm regeneration when applied therapeutically after reperfusion injury.

**Methods:**

I/R of the small intestine was initiated by occluding and reopening the upper mesenteric artery in rats. After 60 min of ischemia and 300 min of reperfusion, glycine, sodium pyruvate, or resveratrol was administered intravenously. Small intestine regeneration was analyzed regarding tissue damage, activity of saccharase, and Ki-67 positive cells. Additionally, systemic parameters and metabolic ones were obtained at selected periods.

**Results:**

Resveratrol failed in improving the outcome after I/R, while glycine showed a partial beneficial effect. Sodium pyruvate ameliorated metabolic acidosis, diminished histopathologic tissue injury, and increased cell proliferation in the small intestine.

**Conclusion:**

While glycine could improve in part regeneration but not proliferation, sodium pyruvate seems to be a possible therapeutic agent to facilitate proliferation and to support mucosal regeneration after I/R injury to the small intestine.

## 1. Introduction

A decrease in intestinal blood flow under 30% leads to a critical shortage of the intestine with blood [1]. This undersupply ends up not only in hypoxia but also in CO_2_-retention. An intracellular acidosis and tissue damage with disastrous consequences were evident [1, 2]. Due to the high metabolism rate, especially in the villi, an early disruption of the mucosa occurs whereas crypts are relatively unaffected [2–5]. In addition, the restoration of intestinal blood flow is an extremely harmful process that leads to systemic inflammation, bacterial translocation, and the release of reactive oxygen species and can culminate in multiple organ failures [6, 7]. On the other hand, as the intestinal mucosa is one of the most rapidly proliferating organs in the human body, this tissue is able in part to regenerate itself after reperfusion [8].

Intestinal ischemia is often caused by a malperfusion of the upper mesenteric artery and is one of the most common gastrointestinal complications following cardiac surgery, although it occurs only with a probability of 0.16% of all cardiac operations [2, 9, 10]. Nevertheless, it is of utmost importance to find an adequate therapy because the mortality is higher than 50% [10]. In a rat model of acute mesenteric ischemia (AMI) glycine, sodium pyruvate and resveratrol have been identified as protective agents in intestinal ischemia/reperfusion (I/R) [11–13]. Thereby, ischemia was induced by occluding the upper mesenteric artery and restoring the blood flow afterwards. All these substances were administered intravenously* (i.v.)* in a prophylactic manner, which means before the onset of ischemia, and during the reperfusion period [11–14]. Regarding the regeneration ability, it must be investigated how these agents influence the process of active regeneration even in consideration of an intended clinical application.

In the present manuscript, we used a rat model for AMI as described previously [11, 13, 15] and firstly analyzed the relationship between histological tissue damage, Ki-67-expression in enterocytes, and different lengths of reperfusion after an ischemic period of 60 min. Afterwards, it was proved whether glycine, sodium pyruvate, and resveratrol can support the regeneration process during the reperfusion period. To avoid the uncertainty that the tested substances influence the early reperfusion injury and not the regeneration itself, the* i.v.* application of the selected agents was started 30 min after the onset of reperfusion until the end of the experiment.

## 2. Methods

### 2.1. Animals

Male Wistar rats (400–470 g) were purchased from the central animal unit of the University Hospital Essen. Animals were kept under standardized conditions of temperature (22,8 ± 1°C), humidity (55 ± 5%), and 12/12-hour light/dark cycles with free access to food and water (ssniff-Spezialdiäten, Soest, Germany). All animals received human care according to the standards of the Federation of European Laboratory Animal Science Association. The experimental protocol was approved by the local committee based on the local animal protection act.

### 2.2. Anesthesia, Analgesia, and Surgical Procedure

Rats were anesthetized with isoflurane (Abbott, Wiesbaden, Germany), treated with 10% ketamine (Ceva, Düsseldorf, Germany) and lidocaine (AstraZeneca, Wedel, Germany) for analgesia, and subjected to surgical procedures as described previously [16]. After placement of the catheters (Smiths Medical International, Hythe, UK) the upper mesenterica artery was occluded using an atraumatic minibulldog. After the ischemic period (I, 60 min), the microvascular clamp was removed and reperfusion (R, 300 min) started. At the end of the experiments, all animals were sacrificed by cardiac incision under deep isoflurane anesthesia.

### 2.3. Study Groups

The animal study was performed with 6 to 8 rats per group. In the first experimental study, sodium chloride (NaCl) solution was infused into the femoral vein at a rate of 7 ml*∗*kg^−1^*∗*h^−1^ during the whole experimental period. After an ischemic period of 60 min, different lengths of reperfusion (0, 30, 60, 120, 240, and 360 min) were tested (resp., 6 animals per group) and compared with rats under normoxic conditions (normoxic control group).

In the second experimental study, glycine, sodium pyruvate, and resveratrol (Sigma-Aldrich, St. Louis, MO, USA) were freshly dissolved in NaCl solution (0,9%, Fresenius Kabi, Bad Homburg, Germany) and filtered through bacterial tight filters; pH was adjusted to 7.4. Prepared solutions were infused into the femoral vein at a rate of 7 ml × kg^−1^ × h^−1^ for a total of 270 min. Infusion started 30 min after the onset of reperfusion until the end of the experiment. An ischemic control group continuously received only NaCl solution during the experimental period; a normoxic control group underwent all surgical procedures, without inducing mesenteric I/R. Experimental groups were compared as shown in Table 1.

### 2.4. Biomonitoring and Assessment of Blood and Plasma Parameters

Arterial pressure was measured continuously via the arterial catheter. Breathing and heart rate were determined every 5 to 10 minutes. The body core temperature of the animals was monitored using a rectal sensor. The temperature was sustained by a thermostated operating table and by covering the rats with aluminum foil.

Blood samples were taken from the arterial catheter using a 2-mL syringe containing 80 i.u. electrolyte-balanced heparin (Radiometer Medical, Brønshøj, Denmark). For each blood sample, animals were injected with equal amount of Ringer solution. Arterial oxygen and carbon dioxide partial pressures (pO_2_, pCO_2_), oxygen saturation, pH, acid-base status, hemoglobin concentration, and hematocrit were assessed with a blood gas analyzer (ABL 715; Radiometer, Copenhagen, Denmark). For determination of electrolytes (Na^+^, K^+^, Cl^−^, and Ca^2+^) and metabolic parameters (lactate, glucose) the blood gas analyzer was equipped with additional electrodes. Blood plasma was obtained by centrifugation (3,000*g* for 15 min at 25°C) and stored at −80°C until its use. To quantify cell injury and in particular liver injury, plasma activities of aspartate aminotransferase and alanine aminotransferase were determined with a fully automated clinical chemistry analyzer (Response 920, Diagnostic Systems International, Greiner).

### 2.5. Determination of Macroscopic Intestinal Injury

Subsequent to the reperfusion period, the complete small intestine was resected and cut into 10 pieces of equal length. A sample of 1 cm of segments 3, 5, 7, and 9 (numbering from* duodenum* to* ileum*) was stored in 10% formalin for histological analysis, respectively. In order to inhibit self-digestion, the intestine was added to Petri dishes containing cold (4°C) NaCl-HEPES-buffer (140 mM NaCl, 20 mM HEPES-[4-(2-hydroxyethyl)-1-piperazineethanesulfonic acid], pH 7.4) on ice for macroscopic analysis and enzymatic tests. Then, the macroscopic injury was determined as described previously [17].

### 2.6. Histopathologic Scoring of the Intestinal Injury

Samples were fixed for at least 24 h in formalin (10%, neutral buffered) and paraffin-embedded cross sections (3 mm) were generated. Samples were cut with a microtome device (thickness 1 *µ*m). Before staining, samples were dewaxed with xylol and dehydrated with alcohol. Samples were stained with hematoxylin-eosin. Histopathologic changes were assessed in a blinded manner. I/R injury to the small intestine was scored on a scale from 0 to 8 in dependence on the Park/Chiu system [18, 19] as described previously [12].

### 2.7. Determination of Tissue Saccharase Activity

Subsequent to the determination of the macroscopic score, each segment of the small intestine was cut in the middle, and the resulting 20 specimens were homogenized in 1 mL cold homogenization buffer on ice as described previously [15]. The following determination of tissue saccharase activity has been described in detail elsewhere [16].

### 2.8. Immunohistochemistry of Ki-67

**Figure 1:**
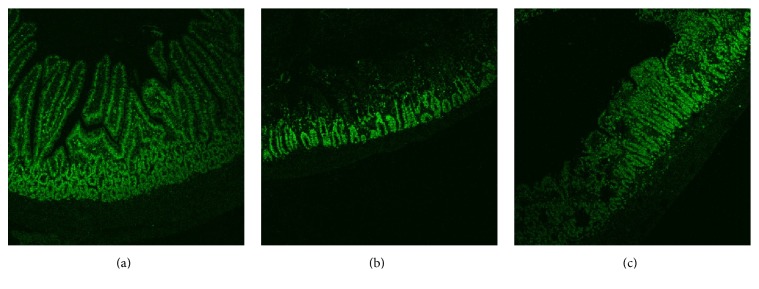
*Ki-67 positive cells after immunohistochemical staining of the rat small intestine*. Pictures (a) to (c) were acquired with a laser scanning microscope, equipped with an argon laser and a Plan-Neofluar ×10/0.30 objective. (a) shows Ki-67 positive cells in the intestinal mucosa under normoxic conditions. (b) shows Ki-67 positive cells after ischemia and (c) the small intestine after 360 min of reperfusion.

After dehydration with alcohol, histological samples were prepared as described above. Ki-67 positive cells were detected using the alkaline phosphatase monoclonal anti-alkaline phosphatase (APAAP) method [20]. The primary antibody against the antigen Ki-67 was diluted 1 : 200 in UltrAb Diluent (Thermo Scientific, Fremont, USA). Hematoxylin was used for counterstaining. So far unknown, this method of staining results in a selective fluorescent signal for Ki-67 positive cells (Figure 1). Therefore, pictures and their quantitative evaluation were performed using a laser scanning microscope (LSM 510, Zeiss, Oberkochen, Germany), equipped with an argon laser and a Plan-Neofluar ×10/0.30 objective. Excitation was from the 488-nm laser line, using emission filters of more than 505 nm. Image processing and evaluation of the Ki-67 pixel fluorescence intensity were performed using the software of LSM 510 imaging system. Data are expressed in arbitrary units (a.u.).

### 2.9. Statistics

Experiments were performed with 6 to 8 animals per experimental group. Power analysis was performed to determine the required sampling size and to prove the statistical significance using the GraphPad Prism 6.00 software (GraphPad Software, Inc., La Jolla, CA). Data are expressed as mean values ± standard deviation (SD). Comparisons among multiple groups were performed by using analysis of variance either for nonrecurring or for repeated measures followed by Fisher LSD honestly significant difference post hoc analysis or Kruskal-Wallis test followed by Dunn's post hoc analysis. A *P* value < 0.05 was considered to be significant.

## 3. Results

### 3.1. Effects of Different Length of Reperfusion on Ki-67 Positive Cells and Intestinal Tissue Damage

To analyze the proliferation rate of the postischemic mucosa, Ki-67 positive cells were quantified by the measurement of their fluorescence intensity after immunohistochemical staining of Ki-67 (Figure 1).

**Figure 2:**
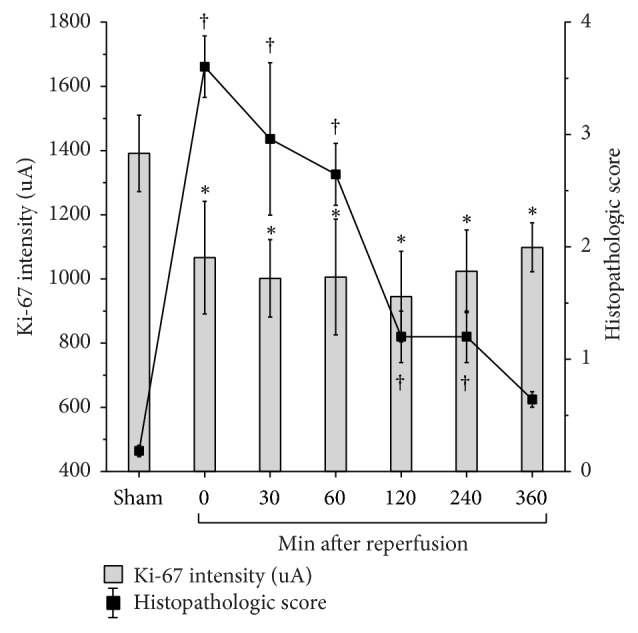
*The mean pixel fluorescence intensity of Ki-67 positive cells and the histopathological score after different reperfusion periods following acute mesenteric ischemia for 60 min in rats*. Data are expressed in arbitrary units (a.u.). Parameters were determined at the end of the respective reperfusion period. Values are means ± standard deviation (SD). Ki-67: ^*∗*^*P* < 0.05 (versus normoxic control group). Histopathologic score: ^†^*P* < 0.05 (versus normoxic control group).

Immediately after ischemia, a significant decrease in cell proliferation could be observed, expressed by a decrease in the mean fluorescence intensity from 1390 down to 1060 a.u. (Figure 2). Until a reperfusion period of 120 min, the intensity further decreased down to 945 and then increased up to 1100 a.u., 360 min after reperfusion. Nevertheless, initial intensity values (1390 a.u.) under normoxic conditions remained significantly higher (Figure 2). The Chiu score was around 0.2 in the normoxic control group and increased significantly up to 3.6 during the ischemic period. Already after a reperfusion period of 120 min, the Chiu score was significantly diminished to around 1.2. Interestingly, after a reperfusion phase of 360 min, there was no significant histopathological difference between ischemic and normoxic rats any more (Figure 2).

### 3.2. Effects of Glycine, Sodium Pyruvate, and Resveratrol

#### 3.2.1. Effects on Arterial Blood Pressure and Other Vital Parameters during Mesenteric Ischemia-Reperfusion (I/R)

**Figure 3:**
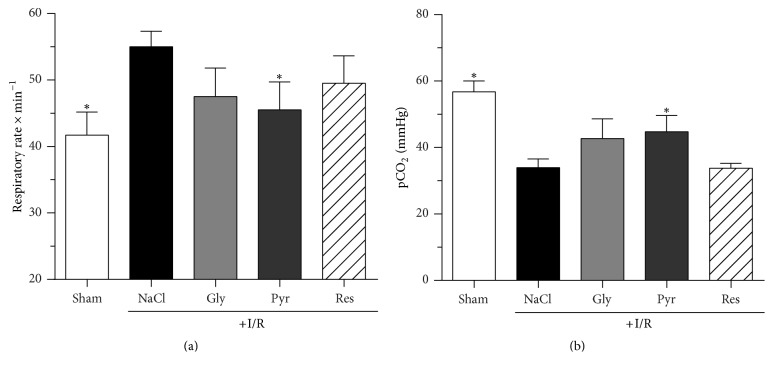
*Effects of glycine, sodium pyruvate, and resveratrol on respiratory rate (a) and arterial carbon dioxide partial pressure (pCO*_2_ *) (b) after mesenteric ischemia-reperfusion (I/R)*. Rats were subjected to mesenteric I/R for 60 min, followed by 300 min of reperfusion. Glycine (15 mg/kg BW × h), sodium pyruvate (50 mg/kg BW × h), or resveratrol (40 *µ*g/kg BW × h) were administered intravenously 30 min after the ischemic period until the end of the observation period. Control animals (±I/R) received the same volume of 0.9% NaCl solution. Parameters were determined at the end of the reperfusion period. Values are means ± standard deviation (SD). ^*∗*^*P* < 0.05 (versus I/R control). Gly: glycine; Pyr: pyruvate; Res: resveratrol; I/R: ischemia/reperfusion.

Induction of mesenteric ischemia caused an abrupt increase in mean arterial blood pressure (MAP; data not shown). During the ischemic period MAP normalized and dropped down during reperfusion. Compared to sham animals, in all I/R groups MAP was significantly lower until 40 min of reperfusion and again 200–290 min after reperfusion had started. Neither glycine, pyruvate, nor resveratrol showed any significant effect on MAP in comparison to the I/R-group. Heart rate was not affected by I/R or any applied substance (data not shown). In contrast, the respiratory rate was significantly lower in the sham group during the reperfusion period intermittently and finally (*t* = 300 min). Remarkably, the animals of the pyruvate group showed a significantly lower respiratory rate than the I/R group that received only NaCl solution (Figure 3(a)).

#### 3.2.2. Effects on Blood pH, Carbon Dioxide Partial Pressure (pCO_2_), Bicarbonate (HCO_3_^−^), Base Excess (BE), Hematocrit (Hct), Lactate, and Electrolyte Concentrations during Mesenteric Ischemia-Reperfusion (I/R)

**Figure 4:**
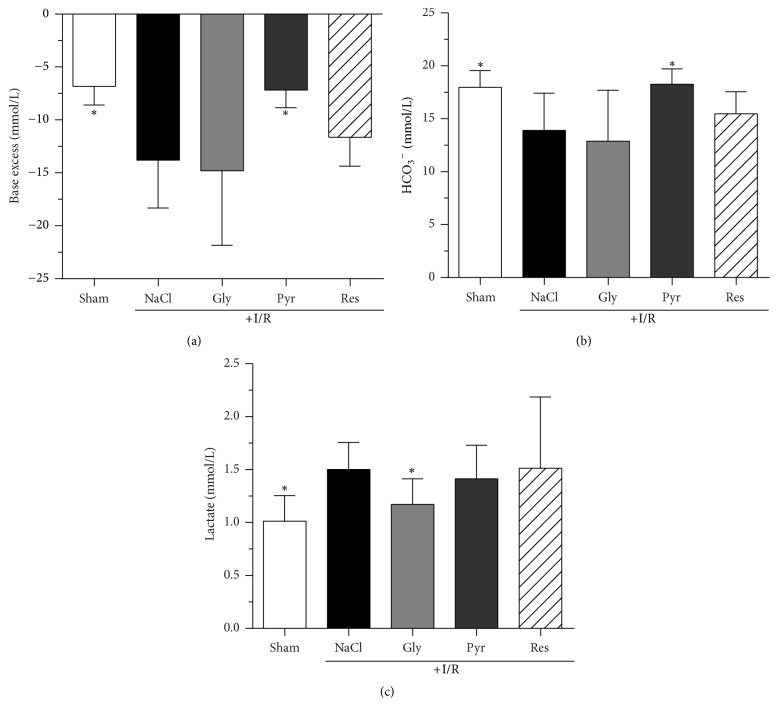
*Effects of glycine, sodium pyruvate, and resveratrol on base excess (a), bicarbonate (HCO*_3_^−^ *; (b)), and arterial lactate concentration (c) after mesenteric ischemia-reperfusion (I/R)*. Rats were subjected to mesenteric I/R for 60 min, followed by 300 min of reperfusion. Glycine (15 mg/kg BW × h), sodium pyruvate (50 mg/kg BW × h), and resveratrol (40 *µ*g/kg BW × h) were administered intravenously 30 min after the ischemic period until the end of the observation period. Control animals (±I/R) received the same volume of 0.9% NaCl solution. Parameters were determined at the end of the reperfusion period. Values are means ± standard deviation (SD). ^*∗*^*P* < 0.05 (versus I/R control). Gly: glycine; Pyr: pyruvate; Res: resveratrol; I/R: ischemia/reperfusion.

During and at the end of the experimental period, plasma electrolyte concentrations of all groups were similar and not effected by glycine, sodium pyruvate, or resveratrol (Table 2). The final hematocrit of the sham group was significantly lower than in I/R group animals (*P* < 0.5) and also not altered by any of the additives. I/R did not change blood pH (Table 2), but the base excess (BE), bicarbonate (HCO_3_^−^) concentration, and carbon dioxide partial pressure (pCO_2_) were changed at the end of the experiment (*t* = 300 min; Figures 3 and 4). The pCO_2_ remained significantly higher in the sham group than in the I/R group (57 ± 7 mmHg versus 34 ± 16 mmHg; *P* < 0,0001) and this I/R-induced decrease was significantly diminished exclusively by sodium pyruvate (45 ± 14 mmHg; *P* < 0,05; Figure 3(b)).

After 300 min of reperfusion, rats of the sham groups showed a BE of −6.8 ± 1.8 mmol/L. In the I/R group BE was clearly lower (−13.6 ± 4.5 mmol/L) and again significantly elevated solely by sodium pyruvate (−7.2 ± 1.7 mmol/L; *P* < 0.005; Figure 4(a)). The final plasma bicarbonate concentration was significantly higher in the sham group (17.9 ± 1.6 mmol/L) than in I/R group rats (13.9 ± 3.5 mmol/L) and, accordingly, only sodium pyruvate normalized the HCO_3_^−^ concentration during reperfusion (18.3 ± 1.5 mmol/L; *P* < 0,005; Figure 4(b)). Intestinal I/R significantly increased the final lactate concentration (1.5 ± 0.3 mmol/L) as compared to the sham group (1.0 ± 0.2 mmol/L). The application of either sodium pyruvate or resveratrol did not show any effect. In contrast, the infusion of glycine (Figure 4(c)) caused a significantly lower blood lactate concentration (1.2 ± 0.2 mmol/L; *P* < 0,005).

#### 3.2.3. Effects on the Small Intestine during Mesenteric Ischemia-Reperfusion (I/R)

**Figure 5:**
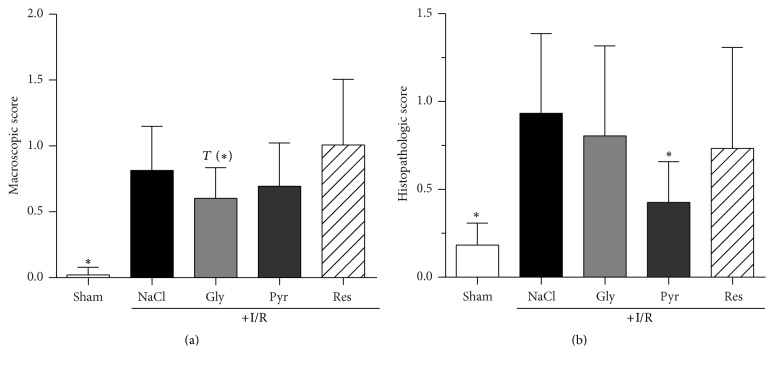
*Effects of glycine, sodium pyruvate, and resveratrol on the macroscopic score (a) and histopathologic (b) score of the rat small intestine after mesenteric ischemia-reperfusion (I/R)*. Rats were subjected to mesenteric I/R for 60 min, followed by 300 min of reperfusion. Glycine (15 mg/kg BW × h), sodium pyruvate (50 mg/kg BW × h), and resveratrol (40 *µ*g/kg BW × h) were administered intravenously 30 min after the ischemic period until the end of the observation period. Control animals (±I/R) received the same volume of 0.9% NaCl solution. Parameters were determined at the end of the reperfusion period. Values are means ± standard deviation* (SD)*. ^**∗**^*P* < 0.05 (versus I/R control). Gly: glycine; Pyr: pyruvate; Res: resveratrol; I/R: ischemia/reperfusion. *T* is the calculated difference represented in units of standard error.

**Figure 6:**
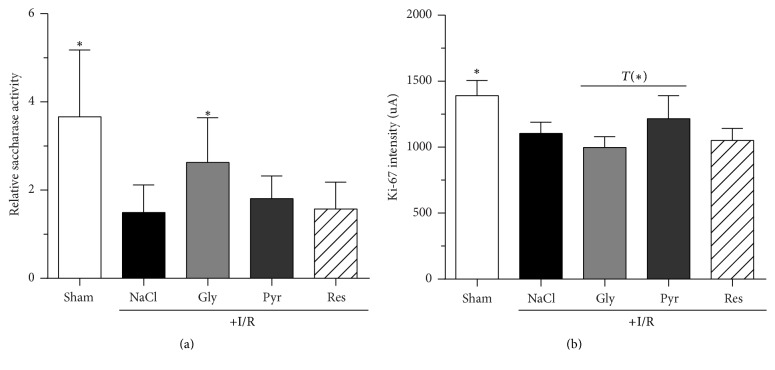
*Effects of glycine, sodium pyruvate, and resveratrol on the relative activity of saccharase (a) and pixel fluorescence intensity of Ki-67 positive cells in the small intestine (b) after mesenteric ischemia-reperfusion (I/R)*. Rats were subjected to mesenteric I/R for 60 min, followed by 300 min of reperfusion. Glycine (15 mg/kg BW × h), sodium pyruvate (50 mg/kg BW × h), and resveratrol (40 *µ*g/kg BW × h) were administered intravenously 30 min after the ischemic period until the end of observation period. Control animals (±I/R) received the same volume of 0.9% NaCl solution. Parameters were determined at the end of the reperfusion period. Values are means ± standard deviation (SD). ^*∗*^*P* < 0.05 (versus I/R control). Gly: glycine; Pyr: pyruvate; Res: resveratrol; I/R: ischemia/reperfusion. *T* is the calculated difference represented in units of standard error.

Ischemia and the following reperfusion significantly impaired the structure and the functionality of the small intestine (Figures 5 and 6). Macroscopic tissue damage was visible on local hemorrhages in the intestinal wall. Thus, the macroscopic score was around 40-fold higher compared to the sham control group (0.8 ± 0.3 versus 0.02 ± 0.06; Figure 5(a)). The application of sodium pyruvate and resveratrol did not improve this outcome, but glycine was able to ameliorate the macroscopic I/R injury clearly (0.6 ± 0.2 *P* < 0.1).

Alterations in the histological structure of the small intestine were still detectable 300 min after reperfusion. Quantified by the Chiu/Park Score [19], the damage was about fivefold higher in I/R group than in the sham group (0.9 ± 0.5 versus 0.2 ± 0.1; *P* < 0.01), while segment 7 (part of the ileum) was the most damaged one (data not shown). The application of either glycine or resveratrol failed in demonstrating any improvement. However, sodium pyruvate diminished significantly the Chiu/Park score to 0.4 ± 0.2 (Figure 5(b); *P* < 0.05).

The functionality of the mucosal brush border was still impaired 300 min after reperfusion, as evidenced based on a 65% decrease in the saccharase activity (Figure 6(a)). Compared to the I/R group, only in the group receiving glycine saccharase activity was it significantly higher at the end of the experiment (1.5 ± 0.5 versus 2.6 ± 0.6; *P* < 0.5).

To quantify cellular proliferation within the intestinal mucosa after reperfusion, the fluorescence intensity of Ki-67 positive cells in histological sections was determined by laser scanning microscopy (Figure 6(b)). In the sham group mucosal fluorescence intensity was significantly higher than in the I/R group after 300 min of reperfusion (1391 ± 108 a.u. versus 1129 ± 116 a.u.; *P* < 0.0001). None of the additives altered the I/R-induced decrease in mean intestinal Ki-67 significantly. However, glycine decreased Ki-67 in segment 7 and tendentially in the whole small intestine, while pyruvate evoked a beneficial effect by trend (1188 ± 123 a.u; *P* < 0.1).

## 4. Discussion

The present study demonstrates that glycine, sodium pyruvate, and resveratrol, known protectants against small intestinal I/R injury, do not hamper mucosal regeneration after AMI in rats. Instead, both glycine and sodium pyruvate improved intestinal regeneration as evidenced by a diminished tissue damage, while sodium pyruvate also somewhat supported cellular proliferation within the mucosa during intestinal reperfusion (Figures 5 and 6).

Our data demonstrate that the postischemic regeneration of the intestinal mucosa started explicitly before cell proliferation (Figure 2); that is, the Park/Chiu score decreased almost two hours before an increased expression of Ki-67 (a nuclear protein relevant for cell proliferation [21]) could be observed. While the histological architecture was nearly reconstructed after six hours of reperfusion, expression of Ki-67 was still lower than under normoxic conditions (Figure 2). This virtual contradiction goes along with results of Park and Haglund who postulated that migration but not cell proliferation is the main indicator for a successful regeneration process [5].

To investigate the effect of glycine, sodium pyruvate, and resveratrol not only on very early cell migration but on the entire regeneration process, we decided to design the present study with a reperfusion period of 300 min after 60 min of ischemia. Overall, I/R led to hypotension during reperfusion, metabolic acidosis with respiratory compensation, increased hematocrit due to a breakdown of the mucosal barrier, intestinal tissue damage, and decreased proliferation of enterocytes. Examining the histological architecture after I/R, nearly healthy areas could be neighboring to areas of complete necrosis and this confusing examination is known to be triggered by the state of differentiation of the villi [22].

Resveratrol (trans-3,5,4′-trihydroxystilbene) is a natural compound of some dietary sources especially red wine [23]. Probably due to its antioxidative and yet unknown properties, resveratrol was found to attenuate I/R injury after AMI when administered in a prophylactic manner [11, 14]. Nevertheless, resveratrol failed to show any beneficial effect on tissue regeneration, when it was administered half an hour after the end of hypoxia.

In previous studies, glycine was found to offer beneficial properties in AMI when infused prophylactically before induction of mesenteric ischemia [12, 24]. More precisely, the amino acid ameliorated macroscopic and histological tissue damage and prevented postischemic shock due to the application of only 10 mg/kg BW of glycine [12]. In the present study, glycine did not stimulate proliferation in the intestinal mucosa but diminished it in the ileum (Figure 6) and any beneficial effect on hypotension was missing. Since there is strong evidence that glycine mediates its prophylactic effect in I/R via the glycine receptor, which is localized not only in the brain but also on immune cells [16, 25], its inhibitory effect on the receptor might play a crucial role in diminishing cell proliferation as well [16]. Nevertheless, glycine showed beneficial effects on the small intestine and might support regeneration but not the proliferation of the intestinal mucosa (Figures 5 and 6). However, whether this impact of glycine turns out to be beneficial or debilitating in the clinical therapy of reperfusion injury remains unsettled, although its prophylactic properties cannot be denied [12, 24].

Recently, we demonstrated that sodium pyruvate is able to diminish I/R-induced injury to the small intestine in the rat [13]. The product of aerobic glycolysis offered effects comparable to glycine and reduced intestinal tissue damage. In addition, a dose of 250 mg/kg BW sodium pyruvate prelimited a metabolic acidosis during AMI, which was getting visible in higher base excess and blood pH [13]. Also, in the present study, sodium pyruvate could prevent metabolic acidosis (Figure 4) and led to an obvious improvement of intestinal tissue damage (Figure 5(b)). In contrast to glycine, sodium pyruvate caused a tendentially higher expression of Ki-67 (Figure 6(b)). Indeed, under anaerobic conditions, pyruvate is converted to lactate, but, during reperfusion, the *α*-keto acid should be metabolized within the citric acid cycle and can provide energy for cell proliferation and the regeneration process.

## 5. Conclusion

The present study demonstrated that only one compound of the three tested harmless agents, that is, sodium pyruvate, seemed to be an adequate candidate to facilitate proliferation after I/R injury of the small intestine even with regard to both a clinical treatment and the support of mucosal regeneration after intestinal ischemia.

## Figures and Tables

**Table 1:** Schema of the experimental groups.

Study group	*n*	Applied Drug^*∗*^ [*x*kg^−1^ BW × h^−1^]	Ischemia/reperfusion period
Sham	6		0 min/0 min
Ischemia/reperfusion, I/R	8		60 min/300 min
Glycine + I/R	8	15 mg glycine	60 min/300 min
Pyruvate + I/R	8	50 mg sodium pyruvate	60 min/300 min
Resveratrol + I/R	8	40 *μ*g Resveratrol	60 min/300 min

Rats were subjected to mesenteric I/R for 60 min, followed by 300 min of reperfusion. Glycine (15 mg/kg BW × h), sodium pyruvate (50 mg/kg BW × h), or resveratrol (40 *µ*g/kg BW × h) were administered intravenously 30 min after the onset of reperfusion until the end of the observation period. Control animals (±I/R) received the same volume of 0.9% NaCl solution. *∗* infused in 7 ml × kg^−1^ × h^−1^ 0.9% NaCl.

**Table 2:** Effects of glycine, sodium pyruvate, and resveratrol on plasma electrolyte concentrations, hematocrit, and pH.

Parameter	^**1**^ **Na** ^**+**^ [mmol/L]	^**2**^ **K** ^**+**^ [mmol/L]	^**3**^ **Ca** ^**2+**^ [mmol/L]	^**4**^ **Cl** ^−^ [mmol/L]	Hematocrit [%]	pH
Sham	140 ± 1.3	5.2 ± 0.5	1.5 ± 0.1	119 ± 2.4	36.1 ± 2.7^*∗*^	7.2 ± 0.1
Ischemia-reperfusion	141 ± 2.8	5.5 ± 0.5	1.4 ± 0.1	125 ± 4.4	41.7 ± 3.6	7.2 ± 0.1
Glycine + I/R	141 ± 1.9	5.6 ± 0.4	1.4 ± 0.1	125 ± 4.4	37.9 ± 7.1	7.1 ± 0.3
Pyruvate + I/R	141 ± 2.6	5.2 ± 0.5	1.4 ± 0.1	121 ± 4.2	40.2 ± 3.5	7.3 ± 0.1
Resveratrol + I/R	141 ± 3.2	5.6 ± 0.4	1.4 ± 0.1	128 ± 5.4	41.7 ± 6.7	7.2 ± 0.1

Rats were subjected to mesenteric I/R for 60 min, followed by 300 min of reperfusion. Glycine (15 mg/kg BW × h), sodium pyruvate (50 mg/kg BW × h), or resveratrol (40 *µ*g/kg BW × h) were administered intravenously 30 min after the onset of reperfusion until the end of the observation period. Control animals (±I/R) received the same volume of 0.9% NaCl solution. Parameters were determined at the end of the reperfusion period. Values are means ± standard deviation (SD). ^*∗*^*P* < 0.05 (versus I/R control).

## Abbreviations

AMI: Acute mesenteric ischemia
I/R: Ischemia-reperfusion
*i.v.*: * Intravenously *
a.u.: Arbitrary units
MAP: Mean arterial pressure
HCO_3_^−^: Bicarbonate
BE: Base excess
pCO_2_: Carbon dioxide partial pressure.
